# The information needs of women diagnosed with Polycystic Ovarian Syndrome – implications for treatment and health outcomes

**DOI:** 10.1186/1472-6874-7-9

**Published:** 2007-06-20

**Authors:** Jodie C Avery, Annette J Braunack-Mayer

**Affiliations:** 1 Discipline of Public Health, The University of Adelaide, SA 5005, Australia

## Abstract

**Background:**

This paper reports the findings of an exploratory study about the information women diagnosed with Polycystic Ovarian Syndrome (PCOS) want to know about their condition and the consequences of this information for future treatment and health outcomes.

**Methods:**

In-depth qualitative interviews regarding their information needs were undertaken with ten South Australian women diagnosed with PCOS. These women were aged 28–38 years and at differing stages of their fertility experience. The time since diagnosis ranged from 1–17 years. The main outcome measures sought were the identification of the information needs of women diagnosed with Polycystic Ovarian Syndrome (PCOS) during different periods of their lives; how and where they obtain this information, and the consequences of this information for future treatment and health outcomes.

**Results:**

The women with PCOS in this study preferentially used the Internet for their information needs, as it had the advantages of convenience, privacy and accessibility, when compared with traditional mechanisms of information provision.

**Conclusion:**

Giving a name to a collection of symptoms may bring relief and provide recognition that there really is a problem. However, with a diagnosis comes the need to have questions answered. A diagnosis of a chronic condition such as PCOS necessitates decision-making regarding possible treatment strategies and lifestyle choices. Information is needed in order to participate in shared decision making. The Internet proved to be a most versatile and beneficial source of information source for women with PCOS, if its limitations are taken into consideration.

## Background

Polycystic Ovarian Syndrome (PCOS), the most common endocrine disorder in women [1], generates numerous health problems, usually commencing at puberty. PCOS affects approximately 5 to 10% of women of reproductive age in Australia [2].

The aetiology of PCOS is unclear: however, genetic inheritance may be implicated [3], and both insulin and androgen production are affected. Women diagnosed with PCOS have an increased risk of menstrual disorders, hirsutism, infertility, miscarriage, obesity, cardiovascular complications, endometrial cancer, and a seven fold greater risk of Type II diabetes [4].

The symptoms of PCOS may appear at any age during the reproductive life, and manifest in some groups of women differently to others. Women with PCOS may not seek assistance until they attempt to conceive, as this may be the first time they have not been under the influence of contraception, which may have masked the symptoms [5]. Symptoms such as obesity and hirsutism which were previously just annoying may get worse, and infertility may be a problem. Studies suggest symptoms vary by ethnicity. A 1999 study of Greek Caucasian women aged 17 to 45 years with PCOS (6.8% overall prevalence, n = 192), reported the prevalence of obesity at 38%, 29% experienced hirsutism with no menstrual disorders and 9.2% had menstrual disorders with moderate to severe hirsutism [6]. In Spanish Caucasian pre-menopausal women aged 18 and over, a study undertaken in 2000 found those with PCOS (6.5% overall prevalence, n = 154) had a prevalence of obesity of 36%, 40% had hirsutism, and 100% had menstrual disorders [7]. However, another 2000 study concerning women in the USA aged 18 to 45 years, white and black with PCOS (overall prevalence of 6.6%, n = 400) had a prevalence of 66% with menstrual disorders only, 19% with hirsutism only, and 14.7% experiencing both [8]. In Iranian girls aged 14 to 18 years, there was an overall prevalence of 3% with PCOS, and of these 6% had hirsutism, 7.4% had menstrual dysfunction, and 4.7% had severe acne [9].

In order to manage its impact, women with PCOS have a great need for information [10]. The physical and psychological problems associated with obesity and infertility, in particular, significantly affect quality of life [11]. Despite the huge increase in the availability of health information concerning PCOS, such as PCOS related books and websites, there has been little research in this area, particularly qualitative research. One of the few studies concerned with general health status of PCOS women examined questions important to women with PCOS. This 1998 study developed a tool to assess the quality of life of women with PCOS, in order to evaluate treatment outcomes [12]. Kitzinger & Willmott concentrated on women's own experience of PCOS within a feminist framework [13]. They found PCOS to be deeply stigmatising, and associated with considerable stress.

There is much consumer information available for many different aspects of health and disease, including PCOS. This information includes that developed by health professionals, advertising disguised as education, and also resources developed by support groups and individuals. The amount of information has increased and has recently become far more accessible with the advent of the Internet. The 1999 South Australian Health Omnibus Survey (n = 3013), a representative survey of people age fifteen years and above [14], found that the sources used to answer health related question in the last twelve months included mostly Doctors (83.6%), with Internet (12.3%) ranked seventh out of ten categories. The same survey found that 77.5% of respondents only sought health information when they had a problem.

Another group specialising in research considering how people use the internet for health research, found that the users appreciated the convenience of being able to seek information at any hour, that they could get a wealth of information online, and that they could do research anonymously.[15]

However, this paucity of research around specific conditions is disappointing. Women with PCOS often deal with their symptoms for a long time, without a specific diagnosis [13]. Many of these women do not know where to look for information. Such women have both a right to more information about their condition and are likely to benefit considerably if information is available. Given questions regarding the reliability, adequacy and agenda behind various sources of potential health information, our hopes are that guidelines can be formulated which assist health care providers in assuring the highest quality of information for the greatest benefit to health consumers.

## Methods

This study used in-depth qualitative interviews to explore the complexity and in-process nature of meanings, attached to PCOS [16]. Women were recruited for the study from two major sources. Initially, seven women were recruited from a previous nutritional study of PCOS, undertaken jointly by the Commonwealth Scientific Industrial Research Organisation's Division of Health Sciences and Nutrition (CSIRO HSN), and the University of Adelaide's Department of Obstetrics and Gynaecology [17]. These women had previously been diagnosed in accordance with the National Institute of Health (NIH) consensus criteria for clinical diagnosis of PCOS [18]. A second group of women were recruited from the Polycystic Ovarian Syndrome Association of Australia (POSAA), a support group for women with PCOS. All women recruited to the study had previously obtained a diagnosis, which provided them with a reference point in their life from which to build their stories.

Intensity sampling, a sampling technique aiming to select cases that manifest the experience being examined intensely, was used to identify potential recruits [19] The characteristics of participants are discussed below.

Women with PCOS were recruited into the study until no new themes were identified during the interviews. Recruitment took place from May to August 2002. Informed written consent was obtained from all participants. Ethics approval was obtained from both the University of Adelaide and CSIRO HSN. Inclusion and exclusion criteria are described in Table 1.

**Table 1:** Inclusion and Exclusion Criteria

**Inclusion Criteria:**	**Exclusion Criteria:**
Female	Non English speakers
Previously diagnosed with Polycystic Ovarian Syndrome – (defined for this study as possessing two or more of these clinical features):	
*Criteria for Clinical Diagnosis of PCOS:*^30^	
• Hyperandrogenism with or without skin manifestations	
• Irregular menses (oligo-ovulation or anovulation)	
• Absence of other androgen disorders (adrenal hyperplasia or tumour)	
• Polycystic ovaries on ultrasonography (not required for diagnosis but extremely prevalent).	

Ten Australian women aged 28–38 years (mean age 32.4) were interviewed. The narrow age group of the participants in this study reflect the time at which fertility is of most concern in a woman's life, as many of the symptoms of PCOS are directly related to fertility, and this is often when women seek diagnosis. The women in this study were at differing stages of their fertility experience. A profile of these women is given in Table 2.

**Table 2:** Profile of Participants

**Pseudonym^a^**	**Age (Years)**	**Diagnosis (Years)**	**Marital Status**	**Education**	**Fertility Status**
Cynthia	28	2	Single	Post school	Trying to conceive (TTC)
Martha	28	2	De facto	University	1 girl
Portia	35	1	Married	University	TTC
Holly	29	4	Married	University	2 boys
Michelle	29	3	Married	Pre matric	TTC, 2 miscarriages
Petra	36	12	Single	Post school	Not tried
Miranda	36	6	Married	Post school	4 miscarriages, adopting
Rebecca	38	4	Relationship	Pre matric	Starting to TTC
Jemima	30	7	Married	Post school	1 girl, 1 boy
Christine	35	17	Married	Post school	TTC, 1 miscarriage

^a^Pseudonyms are used to conceal the identity and preserve the confidentiality of the thoughts and experiences of the participants of this study.

The interviews were carried out by the first author. The first author had previously been involved with a nutrition study, as both a subject and a study coordinator [17] and many of the women were recruited from this same study. Thus, the subjects knew that the author had PCOS, and camaraderie had been established prior to interview. The interviews were reflexive; as the first author had her own experience of the condition, she had been part of the experience of the women in the past, and this experience was the basis of the relationship formed through the interviews such that the subjects and the interviewer were willing to openly exchange their experiences. This also provided the first author with insights during the analysis that may have not otherwise occurred [16].

Interviews were carried out in the women's own homes. The original interview schedule was developed from a review of the literature surrounding PCOS, patient information and education. Questions were developed relating to when the subjects received information, where they obtained it, whether it was helpful, and in what form it came. Other questions were asked regarding the women's own particular experience of the condition, their health status, treatments they had embarked upon, and their own rationale and interpretation regarding the aetiology of the condition. After the piloting, the schedule was modified and, consistent with a grounded approach, it was further developed during the interviews as concepts, categories and themes were identified [16]. The full interview schedule is provided in Table 3. A number of themes from the study are reported on, as they represent stages in the information collection process. These include Information and Diagnosis of PCOS, Information Sources, and Preferences for Information Provision. The time taken for the interviews was between 45 to 90 minutes, and all interviews were tape-recorded.

**Table 3:** Interview Schedule

**Diagnosis of PCOS**
Think back to when you first found out you had PCOS. Tell me about how you were diagnosed?
• When did you find out?
• Who told you?
• Why do you think there was a problem/seek help in the fist place?
• What did they tell you?
• How do you feel about what you found out?
• What happened next?
Could you tell me what you knew about PCOS when you were told that you had it?
• Where did you get that information?
• Who told you where to look for information?
• Were you interested then in finding out more information?
• Have you sought information since then?
• At the time, what kind of information did you want to know?

**Information about PCOS**

What do you understand now about PCOS?
• What do you think causes PCOS?
• What are the symptoms and long term effects?
• What kinds of treatments are there?
• How do you feel about having PCOS now?
What information have you collected about PCOS?
• What have you learnt from this information?
• What did you do with this information
• What did you like/dislike about this information?
• What is the best way of obtaining information?
• What format would you like information presented?

**Outcomes/Lifestyle**

Do you think that the information you've had about PCOS has helped you take better care of yourself? How?
• How do you think your general health will be affected in the future?
• What information have you had about how PCOS affects getting pregnant?
• From this information how do you think PCOS will affect you chance of getting pregnant?
Are there things you would like to know about PCOS for the future?
Considering your life experiences, do you think you look for information in the same way as others?
If you could start over again:
• Who do you think is best to give you information about PCOS?
• Where would you go to look first for information if you had no guidance?
• Why do you think that is a good source?
• What would you do differently about getting your information about PCOS?
Is there anything else you think may be important in order to understand your experience with information about PCOS that we should discuss?

Each of the interviews was transcribed and reviewed by the first author, which enabled further familiarity with the emerging themes of the interviews. 'Framework' content analysis, an analytical process involving a number of distinct yet highly interconnected stages [20], was used to categorise themes for discussion. This method was chosen because of its ability to facilitate systematic analysis. After transcription, data was managed using QSR N6 Student software [21].

## Results and discussion

### Information and diagnosis of PCOS

The participants in this study often began their search for information in an environment of uncertainty.

*I think one of the difficult things about it was because the symptoms are so diverse and I didn't feel like I had some of them. But I definitely had others of them so it was hard to actually work out whether it applied to me or not. And because no one gave me a definite "Yes, you have it", I still was saying well I don't know have I got it, no I haven't, you know, I don't know, this person says I haven't, this person says I have. So what was probably the most important thing was to find out, "Did I have it?" and what constituted the fact that I had it – so that was probably the most pressing thing. (Portia)*.

In order to "have an illness", there needs to be a recognition that problems being experienced are abnormal, and part of a greater disorder. In the case of PCOS, symptoms can be explained by other causes, such as stress, diet, lack of exercise and hormones. The path to diagnosis of an underlying cause is delayed, creating further problems with illness, fertility, state of mind and quality of life. Kitzinger and Wilmott refer to the frustration and anger PCOS women felt about delays in diagnosis, lack of information and an unwillingness of health professionals to take their symptoms seriously [13]. These findings demonstrate the dominant themes that women with PCOS face in their path to diagnosis.

Women suffering from PCOS present their initial problem, with differing symptoms that alone may not be cause for major concern. When associated with other symptoms, the effect is compounded and medical advice may be sought.

I had irregular periods as a teenager. My mother, finally after going from GP to GP, the GP just saying "Teenagers have irregular periods", found another GP who referred me to a gynaecologist, who did some tests, different blood tests, didn't do an ultrasound or anything. When I went back to see him he said, "Bit of a hormonal imbalance, come back and see me if you ever have trouble having children". As an 18 year old I thought "yeah right," and didn't think much more about it. (Jemima)

It was important to the women that someone recognised that they did have a problem. Radley talks about resolving the worry caused by uncertainty about signs and symptoms, by having others establish that one is really ill [22]. This social comparison provides a mechanism for the evaluation of one's symptoms. A medical confirmation of an illness can further reassure suspicions of an illness. In this sense, recognition of a problem validated the women's concerns.

Upset, I was upset when I found out. Relieved at the same time because finally I had a name for something that was wrong with me. (Cynthia)

Prior to obtaining verification of their symptoms, and due to their frustration at the delay in diagnosis, some of participants in this study approached the search for relevant information in a different way. Through past knowledge, gathering of information, and sometimes coincidentally via acquaintances, some of the women suspected that their symptoms did in fact reflect something called Polycystic Ovarian Syndrome, and presented this idea to their doctors.

I was probably self-diagnosed in the end... In between seeing the gynaecologists, doing loads of blood tests, an ultrasound, I was in a bargain basement bookshop, they had a one-dollar book on infertility. I opened it up and looked up irregular periods, read through the thing it said PCOS and I said "Hey I've got that, I've got that, I've got that!" So I went along for my follow up appointment, and he said, "You've got a bit of a hormone imbalance." I said "Have I got PCOS?" and he said, "Yes". (Jemima)

Considering the prevalence of PCOS, surprisingly few participants had knowledge of the condition. Once they were given a name, some completely identified with the condition, after doing some initial research.

I didn't know anything,, never heard of PCOS. When I went to the first endocrinologist I said I'd been putting on weight, noticed hair growth, no periods, skin tags and skin discolouration and after I'd done all the reading, they were all the symptoms for PCOS. So I didn't know anything at first. (Holly)

Sources of information are presented in different ways. The women with PCOS in this study identified a range of sources, and these varied in terms of the women's levels of satisfaction, in terms of quality, their expectations, access and availability.

### Information sources

Most women were diagnosed by general practitioners, gynaecologists or endocrinologists. Their expectation was that they would receive information about their condition through this initial source. Experience of the initial information that doctors gave them was varied. It has been shown that there are marked differences between the different specialties in the diagnosis and management of PCOS [23]. The women considered that some doctors did not know very much about PCOS and most were not satisfied with this information.

*"Some of them have got no idea. I think we know more than them" (Michelle)*.

Kitzinger and Wilmott reported a similar finding, as women who have sought information about their condition upset the doctor-patient relationship balance, complain that they are more informed than the doctors in general, and do not receive positive responses when they suggest treatment options [13]. This finding has been reflected in other similar studies [24,25].

I went to my GP and he sent me on to an endocrinologist, and he told me that with the symptoms that I was telling him that I had, that I probably had PCOS and to go home and lose weight... When I went back to my doctor, he didn't actually know a lot about it. He didn't know a lot about treatments for PCOS. He was very supportive, in our wishes to have children... I'm sure there are doctors out there now who know a lot more about PCOS, I don't know about GPs, I never got a lot of information about it from GPs, but I'm sure it's well known. (Holly)

Doctors focused on fertility issues as the main problems for those with PCOS, often with the worst-case scenario being emphasised. This was evident in both the women who were trying to conceive, and those where this was not yet an issue. The baseline health risks of the syndrome were not emphasised. Some women were left feeling as though they were powerless to do anything about their condition until they wanted children.

I think it's important to ensure that the medical profession are fully aware, both male and female doctors, and have that information on hand, that first port of call for people who need more information and then to have something on hand to make it easier for people to find out information. I felt like the doctor just did not explain it to me properly and I think doctors need to, I mean they do it with everything. If she'd sat there and told me what my major risks were or whatever, I probably would have taken some steps to do things differently. But from this there doesn't apparently to be anything much you can do until you start having children, then it becomes an issue but as far as the doctors are concerned, unless you've got worse symptoms with the hair and those other things, but if you don't have many symptoms, its all just a matter of just wait and see. (Rebecca)

Some of the participants realised that, at diagnosis, the main issues of PCOS were not of great concern to them. This meant that they might only have retained the information that was important to them.

I really can't begrudge the information my doctor gave me, my local GP. I mean, I'm sure that if I had wanted to take it further, she would have been happy to get other references and things for me. Like I said, it wasn't relevant. It was just I had a name. (Martha)

Those that did have positive experiences with their doctors thought that they were fortunate and this kind of experience was rare.

My local GP is very well up on it... The thing I enjoyed about talking to that doctor was that he seemed to know what I was talking about. The symptoms and things like that. He was the one who said to me this is how you feel, this is what's happening and he was spot on. And he didn't make me feel like I was an idiot. That it was all in my head. He was really, really good. (Michelle)

Information at initial diagnosis was sometimes provided in a take home format, such as a photocopied page out of a medical textbook.

All she basically did was give me the name of the condition and a couple of handouts describing what it was. That was it really. (Martha)

Those who hadn't receive pamphlets wanted some written information to aid in their understanding, after the initial information had been taken in. However, pamphlets were also seen as just part of a whole resource package. Pamphlets had a number of advantages, such as being short, portable, local, and providing information on how to find further information.

That little flyer was really good. I liked it because it was Australian. I found out since then that lots of the stuff differs between America and Australia. But also because it was simple and easy and it was something I could give my parents and family, which is really important. It's hard to explain it. (Portia)

Often looking for information might involve a trip to the library, where books and resources can be found. Local libraries however, did not seem to be helpful, and one participant even felt uncomfortable with the idea. In this study, using a library was associated with level of education. The two women who considered the library, or used it, both had a university education. The woman who did not consider this resource had left school before matriculation.

I don't actually go to the library and say "Hello, can you give me PCOS stuff." Not that I should be ashamed of it but if I don't know stuff then I feel a bit embarrassed asking. (Portia)

The information found in books was scarce, outdated or not at a suitable level for the consumer. Occasionally, information could be found in generalist women's health books, but this often referred to PCOS as a condition of "too many male hormones" [26], which may encourage the stigma of this condition. Sometimes the information provided in books was regarded as paternalistic [27], not providing the information needed for shared decision-making.

I never found a book totally on PCOS... Most of the books that were written by the male doctor type, books I thought were quite clinical and didn't tell the whole truth. This is on reflection because at the time you don't know these things. (Jemima)

Dedicated support groups for PCOS are fairly new in Australia. However, one of the participants had sought out information from an American support group via the Internet prior to the establishment of the Australian network. Three of the women interviewed were involved in the Polycystic Ovarian Syndrome Society of Australia (POSAA), established in 1998. Seven of the women had been involved in a clinical trial regarding PCOS, and this provided contact with other sufferers, as well as information and exercise sessions, designed by a fertility clinic [17]. These groups provided the participants with a very positive experience as it was the first time that they had met with other women who had PCOS. This provided the women with opportunities to share experiences with other sufferers, and for finding information regarding their condition on both an individual and a more general level.

It wasn't until I started doing the diet study that I started communicating with other people that had it and all of a sudden you know I came to accept that I had it, and wanted to know a lot more about it... I think the thing that I did that was really good was that I did the CSIRO study, because there doors opened and the information I found. (Cynthia)

It was important for the women to be able to compare themselves with other sufferers. This gave them a frame of reference to rate their own symptoms. Some women came to believe that they were better off than other women with PCOS, which reassured them. The women found that networking with other women provided them with an opportunity to validate their experience, as well as supply them with practical information at their level, from other women with PCOS, and concerned their interests at the time.

I guess I've learnt the most from chatting with other women who've got it, and learning different things like maybe that is related to PCOS when before I thought it wasn't. I thought it was a different problem or whatever... Talking to other people is a huge thing. (Miranda)

Even taking all of the above sources into account, the Internet was the medium of choice for the participants to obtain their information about PCOS, as it had the advantages of other methods as a well as further benefits. However, while the Internet was considered the best way of obtaining information, it was not ideal for everyone. Nonetheless, the women who did not have easy access to the Internet thought that it would be the best way to obtain information, and were keen to use it. Seven of the participants had access to the Internet at home, two at work, and only one did not have any easy access. Five of the participants used the Internet on a regular basis, and four only used it sporadically.

Finally when the Internet was connected in late 1997, that was the explosion when I connected up to the Internet and thought "My God! – look what we've discovered!"... I've got that many bookmarks on the Internet. Tried to just become informed... Online is good,, but I understand that so many people it's not for them. (Jemima)

Although the participants were keen to use the Internet, they had variable responses to the information they found there. Some women found much valuable information, but some were disappointed with the quality, and others were not able to access the information they wanted. Often women found information on the Internet that they would not normally have received, and they shared this with their doctor. The Internet provided the women with evidence that they could present to their doctors, to which they would not otherwise have had access.

I did a lot of research on the Internet and found out a lot about different treatments for women with PCOS. But the treatments were mainly used in the United States, so I had to convince the gynaecologist that I could give it a go. (Holly)

The Internet had enabled communication via chat groups and email lists for some women. This interaction was similar to that found via support groups, yet it could remain anonymous. The participants found these very helpful.

That's the first thing I did was find out all the information and join a few email groups in Australia. I discovered there was a few PCOS groups, so I joined them... I've participated in some chat groups. (Holly)

Using the Internet did, however, require a degree of skill, and the women who were most proficient were more highly educated. In general, the participants were aware that much of the information on the Internet needed to be critically assessed.

I know how to sift though online material... It's a worry when some people present all sorts of witchcraft, heaps of the stuff and a lot of young naïve people do. (Jemima)

Some women also used the Internet in specific ways; obtaining information from support groups that provided email lists, newsletters and question and answer sessions. Information on the Internet was found to be available at different levels of understanding, and this was appreciated.

I think the thing about the Internet is that you don't have to read huge amounts at one time. You can have a little bit and absorb it and then have a little bit more of it. I don't know how you could do that any other way. So you can sort of go at a level when you're ready to. (Portia)

### Preferences for information provision

There are likely to be a number of reasons for the participants' preference for the Internet as an information source. Firstly, frustration at the paucity of information available via traditional mechanisms to women with PCOS, may be one motivating factor for why these women preferred to obtain information via the Internet. Established health and medical information sources may be inadequate for the information that women want about PCOS. Women with PCOS need time to understand the complexities of their chronic condition. The average length of a medical consultation in Australia is 14.8 minutes [28], which may be inadequate for a condition such as PCOS. In addition, women diagnosed some while ago may not want to retell their story and going through tests again every time they change doctors for other opinions, or just to get new information. Many women have also had unpleasant prior experiences, which undermined their confidence in talking to doctors.

Secondly, many of the participants preferred the Internet because it is private and accessible from one's own home, rather than having to make contact with a library or with medical practitioners. Broom (2005) found that men with prostate cancer, had similar experiences, preferring to be anonymous in their pursuit of information. Women with PCOS find it an embarrassing disease and consequently they may seek anonymity when they look for information. They prefer not to discuss their hairiness, obesity and failure to become pregnant with others who would not understand or empathise with their experience. The women realised that they had many choices of information available to them through the Internet, and this did not seem to be daunting.

I like the way I can access the information, that I can do it all of the time, night and day...Because it's accessible, because there's lots of stuff on there, look up more than one thing. Because it's easy for me to use... I can do it privately as well. At least this way I can explore by myself. (Portia)

Finally, women with PCOS used the Internet because it provided them with a sense of control. This included control over the information that they received, the time they received it, the level of the information, and the amount of information. This finding was also reflected in men with prostate cancer by Broom (2005). This continues to upset the power balance between medicine and health consumers, as the women in this study were obtaining their information outside the usual medical sphere, and on their own terms.

Information about PCOS has grown recently. Women with PCOS want the most up to date information at their fingertips, and the Internet makes this information accessible. Women can become familiar with this area of knowledge, and learn exactly where to look, depending on their information needs.

Because there's a lot on there, there's a lot of different groups and different opinions, and you can compare it. Different studies, its not just one person's opinion. (Michelle)

Women's information needs about PCOS evolve as different parts of their lives are emphasised. Frank (1995) refers to people with illnesses telling stories to work out their changing identities, and women with PCOS need to do this at many different points in their life [29].

I came across some real life stories, which weren't really good because all of them had unsuccessful rates of fertility, so it just upset me... I learnt, I'm not alone, its good to know people around you have it. It's also good to hear that there are some people that aren't doing so well and that there's also good to hear the people who are doing well, because you can sort of rate yourself between that and where you're at. (Cynthia)

Many of the women in this study also had a high level of education, which allowed them to make use of more specialized information resources such as electronic databases, and enabled them to venture into unfamiliar areas such as medicine in their search for information. Much of this information was designed for people with medical training, so was sometimes inappropriate for the average woman with PCOS. However, the women in this group were discerning, and were happy that this information was available to them. The women with the least education in this group made the least use of the Internet, and information resources in general, such as reading material. These women preferred talking with a doctor.

As PCOS is a hidden disease, women with PCOS often have no contact with other sufferers. Other recent studies regarding chronic disease information seekers have illustrated a preference for sources other than the Internet [30], such as social networks. Perhaps this is why women with PCOS seem to make use of Internet for their health information, as well as to network with other sufferers. The Internet provides an initial anonymity, an arena to discuss sensitive issues, and a reciprocal support network, that can be further developed once trust is gained.

## Conclusion

Women with PCOS in this study, in addition to routinely using other information sources, preferred using the Internet. The Internet had the advantages that other sources provided, and a number of extra strengths. The Internet opened a world of information to these women, not just that which the doctor provided. It was convenient, private and highly accessible to most, and the information was available at a number of different levels. The women were also aware of the problems with the Internet. They knew that there was some skill involved in searching for and critically assessing information. Often the women would share the information with their doctors, in order to become involved with managing their health. They began to ask the "right" questions, that led to appropriate and efficient answers [31]. The age group of the women in this study reflects the beginning of the Internet literate generation; these women often had training and access to the Internet via their post school education, or at work. However, similar findings have been reported in other health information seeking groups such as older men with prostate cancer, now embracing the Internet also, such that online information provided empowerment, anonymity, but may upset the doctor-patient relationship [24].

There are some limitations to this study. The participants in this study present a group of women with PCOS who were already relatively confident with taking charge of their health care, via being involved in a clinical trial, and/or being involved in a PCOS support group. Other women, not in this environment, or even unaware of the implications of PCOS on their future health, may not be so assured in seeking information. The women in this group also represent a narrow age group, reflecting the age where fertility, rather than the other symptoms of PCOS, may be a major focus. The finding may not be representative of younger women, closer to their primary diagnosis, who may be even more Internet literate. These limitations to the study, particularly the characteristics of the participant group, mean that when discussing information needs, we can only generalise to a similar group of women. Other limitations of this study include the small sample size, and that only one form of data collection was used. The study could be strengthened using triangulation methods, extending to perhaps a questionnaire, and including the women in further discussions of the themes through focus groups. The results of the have also been reported back to participants and members of a PCOS support group.

How then should information be provided to women with PCOS? The medical practitioners may need to be provided with a "sample bag" of information to give to PCOS women at their diagnosis, and follow this information with a review session where the women can ask considered questions about their syndrome. The "sample bag" would need to have pamphlets for a quick run down of the condition and terminology, lifestyle information, support group contact information and a list of reputable web sites; so that further research can be carried out by the woman as her lifestyle and information needs changes over time.

Women with PCOS need to be consulted and involved with creating the design, and assessment of quality of information about their condition, so that this is relevant to their lifestyle. PCOS provides an ideal opportunity for directed health promotion, particularly in terms of diabetes and cardiovascular illnesses, as well as an opportunity for consumer involvement in decision-making, and development of clinical guidelines. The most important treatment that we know of for PCOS is promoting healthy lifestyle as early as possible. Women with PCOS need information and guidance about how to do this to enable them to take control of their own health.

## Competing interests

The author(s) declare that they have no competing interests.

## Authors' contributions

The research reported here was carried out while both the authors were based in the Discipline of Public Health, The University of Adelaide. The research was conducted by JCA for her Masters dissertation under the supervision of AJBM. All authors read and approved the final manuscript.

## Pre-publication history

The pre-publication history for this paper can be accessed here:



## References

[B1] Fenichel P, Gobert B, Carre Y, Barbarino-Monnier P, Hieronimus S (1999). Polycystic ovary syndrome in autoimmune disease. The Lancet.

[B2] Kovacs G, Wood C (2001). The current status of polycystic ovary syndrome. Aust N Z J Obstet Gynaecol.

[B3] Kovacs G, Smith J (2001). A Patient's Guide to the Polycystic Ovary - Its Effects on Health and Fertility.

[B4] Norman RJ, Masters SC, Hague W, Beng C, Pannall P, Wang JX (1995). Metabolic approaches to the subclassification of polycystic ovary syndrome. Fertil Steril.

[B5] Norman RJ, Wu R, Stankiewicz MT (2004). 4: Polycystic ovary syndrome. Med J Aust.

[B6] Diamanti-Kandarakis E, Kouli CR, Bergiele AT, Filandra FA, Tsianateli TC, Spina GG, Zapanti ED, Bartzis MI (1999). A survey of the polycystic ovary syndrome in the Greek island of Lesbos: hormonal and metabolic profile. J Clin Endocrinol Metab.

[B7] Asuncion M, Calvo RM, San Millan JL, Sancho J, Avila S, Escobar-Morreale HF (2000). A prospective study of the prevalence of the polycystic ovary syndrome in unselected Caucasian women from Spain. J Clin Endocrinol Metab.

[B8] Azziz R, Woods KS, Reyna R, Key TJ, Knochenhauer ES, Yildiz BO (2004). The Prevalence and Features of the Polycystic Ovary Syndrome in an Unselected Population. J Clin Endocrinol Metab.

[B9] Hashemipour M, Faghihimani S, Zolfaghary B, Hovsepian S, Ahmadi F, Haghighi S (2004). Prevalence of polycystic ovary syndrome in girls aged 14-18 years in Isfahan, Iran. Horm Res.

[B10] Lobo RA (2001). Priorities in polycystic ovary syndrome. Med J Aust.

[B11] Coffey S, Mason H (2003). The effect of polycystic ovary syndrome on health-related quality of life. Gynecol Endocrinol.

[B12] Cronin L, Guyatt G, Griffith L, Wong E, Azziz R, Futterweit W, Cook D, Dunaif A (1998). Development of a health-related quality-of-life questionnaire (PCOSQ) for women with polycystic ovary syndrome (PCOS). J Clin Endocrinol Metab.

[B13] Kitzinger C, Willmott J (2002). 'The thief of womanhood': women's experience of polycystic ovarian syndrome. Soc Sci Med.

[B14] Wilson D, Wakefield M, Taylor A (1992). The South Australian Health Omnibus Survey. Health Promotion Journal of Australia.

[B15] Fox S, Raine L The online health care revolution:  How the web helps Americans take better care of themselves. http://www.pewinternet.org/reports/toc.asp?Report=26.

[B16] Rice PL, Ezzy D (1999). Qualitative Research Methods - A health focus.

[B17] Moran LJ, Noakes M, Clifton PM, Tomlinson L, Galletly C, Norman RJ (2003). Dietary composition in restoring reproductive and metabolic physiology in overweight women with polycystic ovary syndrome. J Clin Endocrinol Metab.

[B18] Zawdaki J, Dunaif A, Dunaif A, Givens J, Hasletine F, G M (1992). Diagnostic criteria for polycystic ovarian syndrome:  Towards a rational approach.. Polycystic Ovarian Syndrome Current Issues in Endocrinology and Metabolism.

[B19] Quinn Patton M (1990). Qualitative Evaluation and Research Methods.

[B20] Ritchie J, Spencer L, Bryman A, Burgess R (1994). Qualitative data analysis for applied policy research. Analysing Qualitative Data.

[B21] QSR (2002). NUD*IST.

[B22] Radley A (1994). Making Sense of Illness:  The social psychology of health and disease.

[B23] Cussons AJ, Stuckey BG, Walsh JP, Burke V, Norman RJ (2005). Polycystic Ovarian Syndrome:  Marked Differences Between Endocrinologists and Gynaecologists in Diagnosis and Management. Clinical Endocrinology.

[B24] Broom A (2005). Virtually he@lthy: the impact of internet use on disease experience and the doctor-patient relationship. Qual Health Res.

[B25] Ferguson T, Frydman G (2004). The first generation of e-patients. Bmj.

[B26] Cabot S (1991). Don't Let Hormones Ruin Your Life.

[B27] Coulter A, Entwistle V, Gilbert D (1999). Sharing decisions with patients: is the information good enough?. Bmj.

[B28] Britt H, Valenti L, Miller G (2002). Time for care. Length of general practice consultations in Australia. Aust Fam Physician.

[B29] Frank A (1995). The Wounded Storyteller:  Body, Illness and Ethics.

[B30] Raupach JC, Hiller JE (2002). Information and support for women following the primary treatment of breast cancer. Health Expect.

[B31] West C (1984). Routine Complications - Troubles With Talk Between Doctors and Patients.

